# Human hepatic gene expression signature of non-alcoholic fatty liver disease progression, a meta-analysis

**DOI:** 10.1038/s41598-017-10930-w

**Published:** 2017-09-27

**Authors:** Maria Ryaboshapkina, Mårten Hammar

**Affiliations:** 1 Cardiovascular and Metabolic Diseases, Translational Sciences, Innovative Medicines and Early Development Biotech Unit, AstraZeneca, Pepparedsleden 1, Mölndal 431 83 Sweden; 2 Cardiovascular and Metabolic Diseases, Translational Sciences, Innovative Medicines and Early Development Biotech Unit, AstraZeneca, Pepparedsleden 1, Mölndal 431 83 Sweden

## Abstract

Non-alcoholic fatty liver disease (NAFLD) is a wide-spread chronic liver condition that places patients at risk of developing cardiovascular diseases and may progress to cirrhosis or hepatocellular carcinoma if untreated. Challenges in clinical and basic research are caused by poor understanding of NAFLD mechanisms. The purpose of current study is to describe molecular changes occurring in human liver during NAFLD progression by defining a reproducible gene expression signature. We conduct a systematic meta-analysis of published human gene expression studies on liver biopsies and bariatric surgery samples of NAFLD patients. We relate gene expression levels with histology scores using regression models and identify a set of genes showing consistent-sign associations with NAFLD progression that are replicated in at least three independent studies. The analysis reveals genes that have not been previously characterized in the context of NAFLD such as HORMAD2 and LINC01554. In addition, we highlight biomarker opportunities for risk stratification and known drugs that could be used as tool compounds to study NAFLD in model systems. We identify gaps in current knowledge of molecular mechanisms of NAFLD progression and discuss ways to address them. Finally, we provide an extensive data supplement containing meta-analysis results in a computer-readable format.

## Introduction

Non-alcoholic fatty liver disease (NAFLD) is the most common chronic liver disease in industrialized countries and a frequent comorbidity of type 2 diabetes and obesity^1^. NAFLD is often used as an umbrella term for conditions ranging from simple steatosis (SS; accumulation of fat in the liver without inflammation) to advanced cirrhosis. Non-alcoholic steatohepatitis (NASH) is regarded either as an independent disease or as a stage succeeding SS in NAFLD progression. NASH is associated with particularly poor long-term prognosis^2^. Some patients with SS never develop NASH or cirrhosis. The progression to end-stage liver disease can take decades^3^, but outcomes for individual patients are tragic. NASH is the second most common and the most rapidly increasing cause of hepatocellular carcinoma (HCC) in patients awaiting liver transplant in the USA^4,5^. Furthermore, NAFLD is a risk factor for cardiovascular disease, chronic kidney disease, extrahepatic cancers and endocrinal disorders^6^.

NAFLD can progress without clinical manifestations for many years. The symptoms can be unspecific (for example, fatigue, elevated liver injury markers). The diagnosis is typically established though liver biopsy and exclusion of other causes of liver disease. The treatment is centered on management of comorbidities (life style modification, weight loss, antidiabetic medication)^7^. Presently, no drugs are approved by the American agency for Food and Drug Administration (FDA) for treatment of NAFLD. Safe and effective medication and noninvasive biomarkers that could distinguish patients at risk of progression to advanced disease are urgently needed^8^. The challenges in clinical practice are closely related to issues in basic research. The molecular mechanisms of NAFLD progression are poorly understood. The patient population is very heterogeneous. Small numbers of patients in many human studies limit the power to detect associations. As a consequence, basic research on NAFLD progression is plagued by sporadic observations, point-wise hypothesis testing and extensive use of animal models, which may not capture all relevant aspects of disease dynamics in humans^9^.

A reproducible gene expression (mRNA) signature of NAFLD progression could improve our understanding of the disease and help to identify candidate biomarkers or drug targets. The aim of our study is to obtain such signature in adult human liver. Longitudinal liver biopsies are hard to obtain because of ethical reasons and risk of complications. Histological manifestations such as inflammation or fibrosis reflect the severity of NAFLD. Ordering patients in a cross-sectional study by a given histology score from none to mild to severe results in a pseudo time course of disease progression. Hence, genes associated with severity of histological manifestations build up the signature of NAFLD progression. This is the main idea behind the design of our study. We perform a systematic meta-analysis of microarray experiments on liver tissue of NAFLD patients. We relate mRNA levels to histological features using regression models, identify associations that are replicated in at least 3 independent cohorts and combine genes associated with distinct histology scores into the final signature (presented in heatmaps in the Results section).

**Figure 1:**
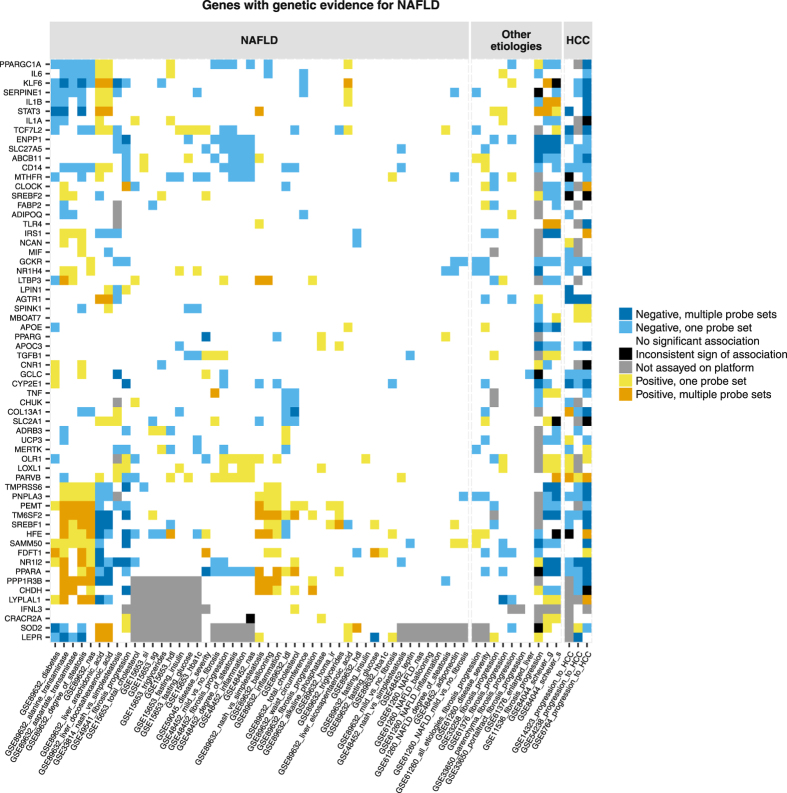
Association profiles between mRNA levels of genes with genetic evidence with NAFLD and histological and biochemical characteristics of the patients. Associations are clustered along x-axis based on their similarity across genes. Associations are separated into three blocks based on disease etiology: NAFLD, other etiologies (viral, parasitic or mixed etiology liver disease), progression towards HCC (abbreviated as HCC). Genes are clustered along y-axis according to similarity of associations of their mRNA levels with histology scores and biochemical traits in NAFLD. Presence or absence of a significant association and sign of each association are colour-coded.

**Figure 2:**
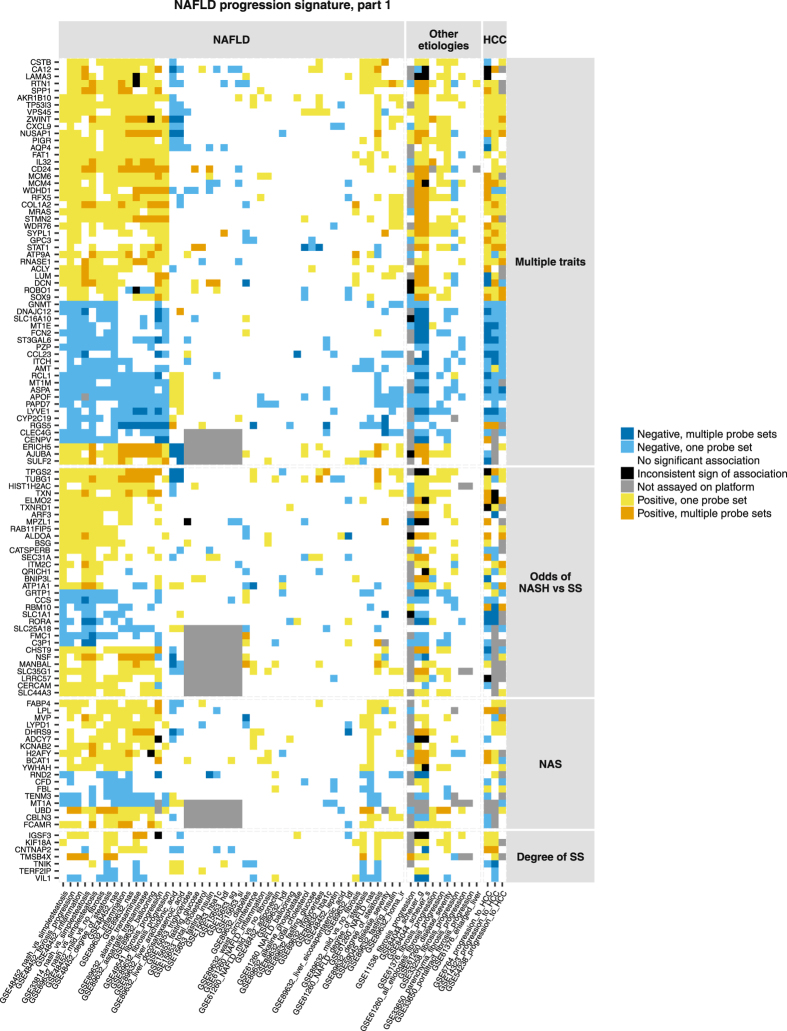
Human NAFLD progression signature (part 1). Figure shows genes with high confidence associations (consistent-sign associations replicated in 3 independent studies) with multiple histological traits, odds of NASH versus SS, NAS score or degree of SS. Genes can also display consistent-sign associations with related histological traits. Genes and associations are organized along x and y axes by the same principle as in Fig. 1.

**Figure 3:**
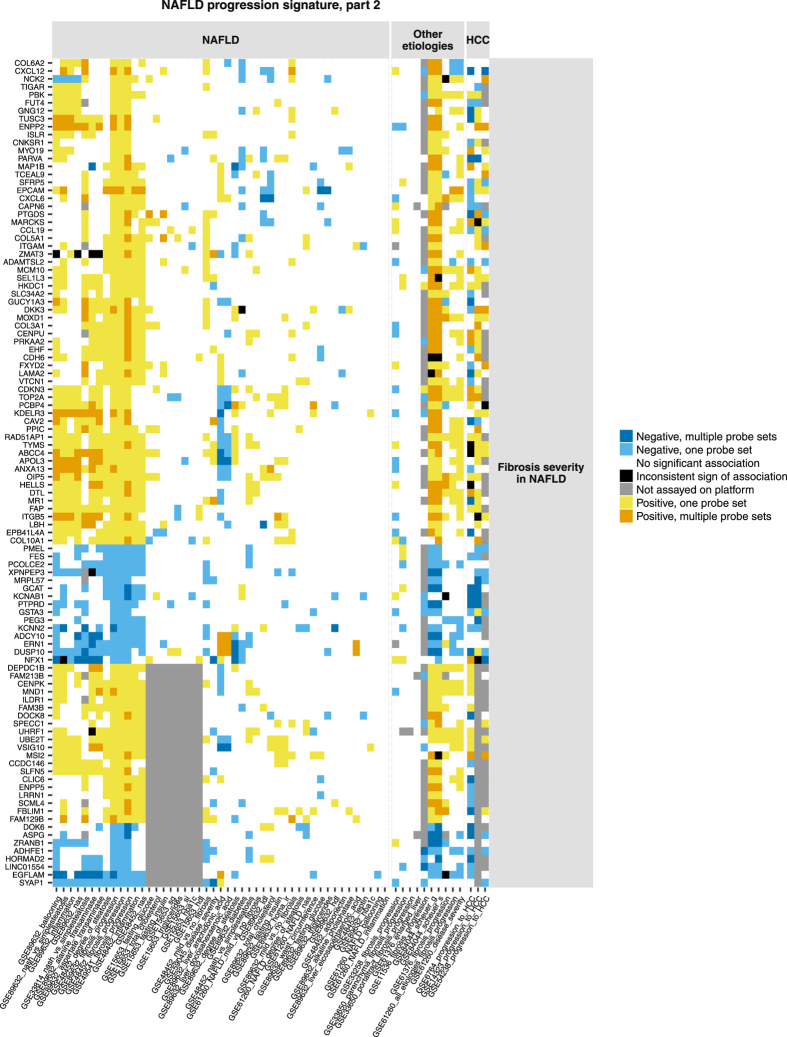
Human NAFLD progression signature (part 2). Figure shows genes with high confidence associations (consistent-sign associations replicated in 3 independent studies) with progression of NAFLD-induced fibrosis. Genes can also display consistent-sign associations with related histological traits. Genes and associations are organized along x and y axes by the same principle as in Fig. 1.

## Materials

### Gene expression data sets

We searched Gene Expression Omnibus (GEO)^10^, ArrayExpress^11^ and Sequence Read Archive^12^ for studies on NAFLD progression in humans and selected GSE48452^13^, GSE61260^14^, GSE89632^15^, GSE59045^16^, GSE49541^17^, GSE15653^18^ and GSE33814^19^. The studies had at least 15 liver biopsies or samples from bariatric surgery patients. NAFLD was established histologically. Each study either included samples from different stages of NAFLD or contained publicly available individual-patient level information on histology traits, liver injury markers or diabetes-related traits. Histological characteristics for patients in GSE61260 were obtained from the corresponding methylation experiment GSE61258^14^. We also identified data sets with histologically scored fibrosis in chronic hepatitis C (GSE33258^20^, GSE33650^21^ and GSE11536^22^), chronic hepatitis B (GSE84044^23^), and fibrosis in chronic mixed viral or parasitic infection (GSE61376^24^). Three data sets covered progression from normal liver to viral hepatitis-induced HCC (GSE6764^25^, GSE54238^26^ and GSE14323^27^) and acted as surrogate material for progression towards NAFLD-induced HCC. Preprocessed gene expression data and sample annotation were obtained from GEO (Series Matrix). Preprocessed data had been quality controlled, background corrected and normalized by the authors of the respective original publications. These data were ready-to-use for downstream analyses. For example, biological samples in GSE48452 were prepared and mRNA extraction was performed according to the standard manufacturers protocols for HuGene 1.1 ST arrays^13^. Ahrens *et al*.^13^ normalized the arrays with RMA method using R package oligo (from sample description on GEO). We mapped internal microarray platform identifiers to NCBI Gene identifiers (Entrez IDs) using annotation included in the data sets and HUGO gene nomenclature committee data^28^. Entrez IDs were subsequently used to integrate results of regression and co-expression analyses between experiments.

### Targets of marketed and clinical trial drugs

Mechanism-of-action human protein targets of marketed and clinical trial drugs were retrieved from ChEMBL version 22^29^. UniProt identifiers were mapped to Entrez IDs and gene symbols using complete human proteome information downloaded from UniProt website on 28.12.2016^30^.

### Data for identification of candidate biomarkers

Genes encoding predicted secreted proteins and proteins with preferential expression in liver (categories ‘Tissue enriched’ and ‘Tissue enhanced’) were identified in Human Protein Atlas data available at www.proteinatlas.org on 29.12.2016^31^.

### Genes with genetic evidence for NAFLD

We focused on genes that had been reviewed by Wood *et al*.^32^ as well as MBOAT7^33^ and MERTK^34^ associated with NAFLD severity.

## Methods

We provided a detailed explanation of properties of the data and the statistical basis behind the approach in Supplementary Methods. Here, we outlined key features of the analysis and described methods for visualization of results.

### Regression analysis

mRNA expression was measured as normalized log_2_-scale fluorescence on a probe set, i.e., a cluster of sequences targeting a given gene. A gene could be represented by a single or multiple probe sets depending on microarray design. Individual data sets quantified histological features differently (e.g., percent of steatosis in GSE89632 vs steatosis score in GSE61260) and were assayed on unrelated platforms (see Table S7 in Supplementary Methods). Merging data sets and obtaining pooled estimates was inappropriate. Every data set was analysed separately. Each probe set was tested for association with disease severity, histology and biomarker traits using linear or logistic regression as summarized in Table S1 in Supplementary Data. Models were adjusted for the most likely sources of variation (e.g., BMI) when the information was publicly available and sample size permitted estimation of a multivariate model. Two-sided p-values below 0.05 were considered significant (H_0_: regression coefficient for mRNA level = 0). Regression coefficients and p-values were rough (limited sample size and linear regression as a simplified model for scores) and had different quantitative interpretation (linear vs logistic regression, covariate structure). We extracted information with compatible meaning for all models: presence/absence and sign of association. All models tested null hypotheses of no association between mRNA and a given aspect of NAFLD progression. All outcome variables were encoded so that low values indicated mild disease and high values indicated severe disease. Sign of regression coefficients always showed direction of association, i.e., increase or decrease of mRNA levels with NAFLD progression. Sign error was the least probable error type in our analysis settings (Supplementary Methods, section ‘Robustness of regression analysis with respect to null hypothesis test of no association and estimated direction of regression slope’).

### Derivation of NAFLD progression signature

Replication in independent studies was used to control false positive discoveries (Supplementary Methods, section ‘The role of replication in independent studies’) as an alternative to Benjamin-Hochberg correction for multiple testing within a single experiment. Genes, whose mRNA levels showed consistent-sign associations with a trait in at least three independent data sets, were considered high confidence observations and formed the NAFLD progression signature (marked as ‘main’ in Table S1 in Supplementary Data). The criterion for replication in 3 studies was motivated by data availability (state December 2016) but sufficient to fulfill its purpose (see Supplementary Methods, section ‘The role of replication in independent studies’). Associations with other traits, which did not live up to the ‘high confidence’ definition, did not directly contribute to the signature and were not used as selection criteria for the genes. Such associations provided additional supportive evidence and were used for visual examination of results (marked as ‘negative control’ and ‘sanity check’ in Table S1 in Supplementary Data, explained in dedicated sections of Supplementary Methods). If associations between a gene and a trait (e.g., fibrosis) were detected on multiple probe sets, all associations were required to have consistent sign (within-study replication).

### Clustering

All associations for a given gene were organized in a fingerprint with 5 possible values: not assayed on microarray platform (either no probe sets targeting a given gene or probe sets with ambiguous mapping to two or more genes), no significant association, association with inconsistent sign for different probe sets, positive or negative association. Complete-linkage hierarchical agglomerative clustering (default hclust implementation in ref.^35^) was based on modified Hamming distance. Distance (D) between fingerprints was defined as:

$$D=1-\frac{\sum _{i=1}^{N}weight}{N\ast 2}$$

where i was the i^th^ position in the fingerprint and N denoted the total number of associations. Weight could take three values. Weight equaled 1 if both genes were not assayed in an experiment or had no associations with trait or inconsistent-sign associations with a trait. Weight equaled 2 if both genes had same-sign associations with a trait. Weight was zero in all other cases.

### Meta-network construction

We constructed co-expression networks from NAFLD data sets GSE48452, GSE61260, GSE49541, GSE89632 and GSE33814. GSE59045 and GSE15653 had 15 and 18 samples respectively and were too small to be included in this analysis. In each data set, Spearman correlation coefficients were computed for all pairs of probe sets representing genes in NAFLD progression signature and genes with genetic evidence for NAFLD. Gene level correlations were obtained by averaging correlation coefficients across all pairs of probe set for a pair of genes. For example, gene A was represented by probe sets a1 and a2 and gene B by probe set b in study X. Then, correlation between A and B in X was mean correlation between (a1 and b) and (a2 and b). Threshold >=0.53 on absolute scale was chosen as the smallest cut-off value that resulted in approximate scale-free topology in 4 out of 5 individual networks (model fit for ‘scale-freeness’ with R^2^ >= 0.8, pickHardThreshold method in WGCNA package^36^, details in Supplementary Methods, section ‘Notes on the meta-network’). Correlations reproduced in >=3 of 5 individual networks constituted the meta-network. Meta-network construction was motivated by heterogeneity of biological material and suboptimal sample size^37^.

### Software

All analyses were performed in R version 3.2.5^35^. Data sets and sample annotation were retrieved using GEOquery package^38^. Networks were created using igraph package^39^. Figures were produced with ggplot2^40^ and ggnetwork^41^ packages.

### Data availability statement

All data sets analysed in the current study are available from Gene Expression Omnibus (https://www.ncbi.nlm.nih.gov/geo/). All data generated during this study and data behind the figures are included in this published article and its Supplementary Data.

## Results

### Genes with genetic evidence for NAFLD

Genetic evidence refers to NAFLD-associated single nucleotide polymorphisms (SNP), i.e., point variants of genomic DNA in immediate vicinity of a given gene or within the gene. Genes with genetic evidence can predispose a patient to develop NAFLD or contribute to disease progression when the patient has at least one copy of risk allele of the SNP. Such genes represent ‘weak spots’ in liver biology and form a special category of interest for our analysis because studying their mRNA expression in NAFLD (regression analysis) and relationships to the signature genes (meta-network) might provide additional insights into NAFLD biology.

The patients in each study represented random samples from the underlying population. Genotypes of patients were unknown. mRNA expression was not allele-specific. Hence, we did not expect a consistent association pattern in all studies. We detected three fairly well defined gene communities by association profile (Fig. 1). Genes related to lipid metabolism FDFT1, PNPLA3, SREBF1 and TM6SF2 clustered together with TMPRSS6, NR1I2 (also known as PXR), SAMM50, HFE and PEMT. Their increasing mRNA levels related to increase in liver injury markers, worsening of steatosis and inflammation as well as decrease in intrahepatic levels of arachidonic and docosahexaenoic acids in GSE89632. An opposite-sign relationship was observed for IL1A, IL1B, IL6, KLF6, PPARGC1A, SERPINE1, STAT3 and TCF7L2. Decreasing expression of ABCB11, CD14, ENPP1, MTHFR and SLC27A5 accompanied fibrosis progression in GSE49541 and/or GSE48452. Decreasing mRNA levels of AGTR1, GCKR, GCLC, CD14, CYP2E1, NR1I2, PPARA, PNPLA3 and TM6SF2 and increasing expression of PARVB were associated with progression from normal liver to HCC (high confidence observations).

### Signature of NAFLD progression

In total, 218 genes showed high confidence associations with at least one histology aspect of NAFLD progression (Figs 2 and 3). The signature genes were unlikely to represent chance findings (Table 1, details of permutation experiment in Supplementary Methods, section ‘The role of replication in independent studies’). Genes associated with fibrosis severity in NAFLD tended to have same-sign associations with fibrosis in hepatitis B but not in hepatitis C (Fig. 3, same-colour columns in GSE84044 and GSE61376 versus predominantly white columns in GSE33650 and GSE33258). We observed no clear separation between gene sets related to distinct aspects of liver histology. The associations were often complemented with supportive same-sign evidence for related traits. For example, elevated mRNA levels of SPP1 (osteopontin) were associated with increasing NAS and inflammation (high confidence observations) as well as with increasing degree of SS in two studies and higher odds of NASH over SS in two studies (Fig. 2). Inflammation and SS were components of NAS score^42^.

**Table 1:** Number of signature genes compared to number of chance findings in random permutations of the data.

Category	Consistent-sign association in 3 independent studies with …					
	…at least 1 trait (any)	… multiple traits	… odds of NASH vs SS	… NAS	… degree of SS	... fibrosis severity in NAFLD
N genes in NAFLD-progression signature	218	57	32	18	7	104
N genes in 1,000 random permutations, median (95% CI)	3 (0–14)	0 (0–0)	0 (0–4)	0 (0–5)	0 (0–4)	0 (0–9)

The analysis confirmed 98 genes that were highlighted in the original publications in the context of NAFLD, hepatitis with other etiologies or progression to HCC (Table S3 in Supplementary Data). Some prominent examples included UBD (ubiquitin D), GPC3 (glypican 3, a gene investigated as diagnostic marker and drug target for HCC^43^), genes involved in collagen life cycle or associated with risk of cirrhosis^44^: CD24, COL1A2 and COL3A1, CXCL6, DCN, EHF, FAP, LUM, PCOLCE2 and SOX9. ACLY, a key enzyme responsible for the synthesis of acetyl coenzyme A, has been described by Ahrens *et al*. as a candidate epigenetic driver of NAFLD^13^. AKR1B10, an enzyme converting aldehydes to alcohol and whereby carrying out a detoxication function, has been identified through differential expression between steatohepatitis and SS patients by Starmann *et al*.^19^ and by Ahrendt *et al*.^15^ Starmann *et al*. extensively studied expression of AKR1B10 in hepatocytes and suggested it as a promising biomarker of NASH and progression to HCC. GNMT has been identified through differential expression between patients with mild and advanced NAFLD-induced fibrosis by Moylan *et al*.^17^. The enzyme catalyses synthesis of sarcosine from glycine and plays a role in liver detoxication. Gnmt knock-out mice are experimental hepatitis models^45^.

By contrast, the signature contained multiple genes with poorly studied biological function in liver such as CBLN3, CERCAM, ERICH5, GRTP1, HORMAD2, LINC01554, MANBAL, MOXD1, MYO19, LRRC57, SEL1L3 and SLC44A3. Increasing expression of SEL1L3 was associated with severity of fibrosis in NAFLD. An intron variant rs959903 in SEL1L3 has been reported in association with ballooning severity^46^. Decreasing expression of LINC01554 (also known as C5orf27 or FLJ38821) was associated with advancing fibrosis in NAFLD patients. The transcript ENST00000436592.5 corresponding to LINC01554 is polyadenylated according to GENCODE consortium polyadenylation data^47^. LINC01554 could be reliably measured with microarray and polyA+ protocol. LINC01554 is preferentially expressed in liver according to GTEx consortium data (http://www.gtexportal.org/home/)^48^. LINC01554 has been previously described in relation to survival of esophageal cancer patients^49^. HORMAD2 is preferentially expressed in testis and liver^31^. The gene is related to cancer^50^ and has genetic associations with immune diseases such as IgA nephropathy^51^ and inflammatory bowel disease^52^. These genetic associations were identified through GWAS Catalog^53^. In our meta-analysis, expression of HORMAD2 decreased with advancing fibrosis in NAFLD.

### NAFLD progression meta-network

**Figure 4:**
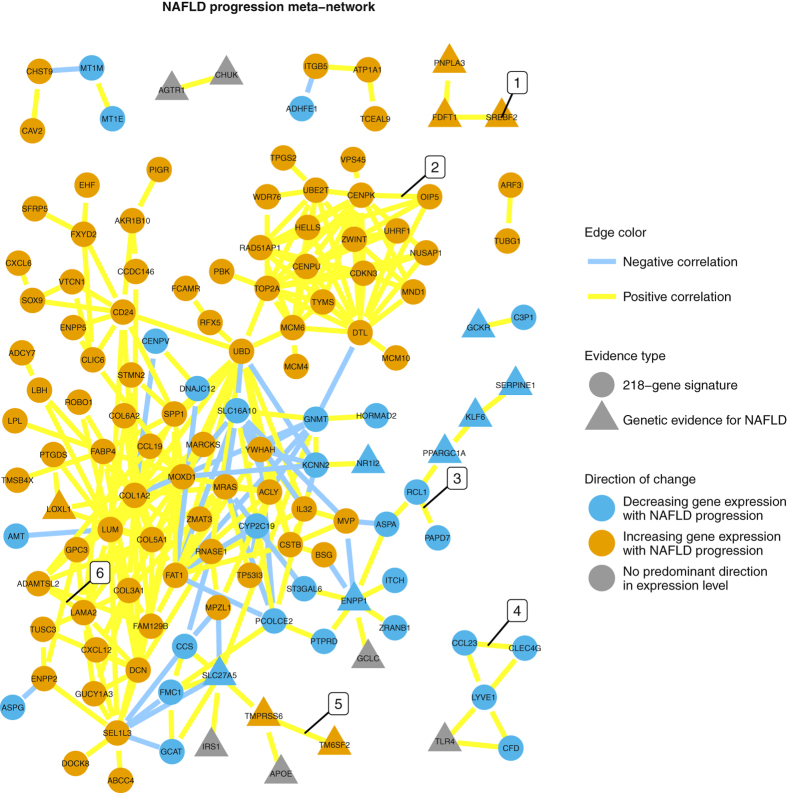
NAFLD progression meta-network. Each node represents a gene. Each edge represents a Spearman correlation between mRNA levels of two genes. An edge is displayed only if the magnitude of correlation coefficient is >=0.53 on absolute scale and the correlation is reproduced in at least three data sets. Lengths of edges vary to improve readability and do not carry mathematical or biological meaning. Six gene communities are highlighted: (1) three genes with genetic associations with NAFLD and role in lipid metabolism, (2) genes involved in cell division, replication and DNA repair, (3) three immune system-related genes with genetic evidence for NAFLD and a ‘bridge’ of genes with enzymatic activity linking them to community 6, (4) putative liver sinusoid endothelium co-expression module^65–67^, (5) genes with genetic evidence for NAFLD and related to fatty acid metabolism, (6) community of genes formed by a large subset of the 218-gene signature and enriched in genes associated with fibrosis. Functional description is provided according to NCBI Gene summary information unless explicitly indicated otherwise.

Among 218 genes in the NAFLD progression signature and 62 genes with genetic evidence, 131 (46.8%) genes had reproducible correlations with each other that satisfied criteria for meta-network construction (Table S4 in Supplementary Data). Genes that clustered together based on their associations with histological traits tended to be correlated. Genes with genetic evidence for NAFLD tended to be co-expressed (communities 1, 3 and 5 in Fig. 4). Genes in the NAFLD progression signature were arranged in three well-formed co-expression modules (2, 4 and 6) and a number of smaller disconnected components. Ten genes with highest number of connections were COL1A2 (25 direct network neighbours), LUM, UBD, DTL, FAT1, MOXD1, CENPK, MRAS, SEL1L3 and TOP2A (12 direct neighbours).

### Drug targets

Among genes with genetic evidence for NAFLD and genes in the progression signature, 21 genes were targeted by marketed and clinical trial drugs (Table 2). Obeticholic acid^54^ and angiotensin II antagonists^55^ are actively investigated in liver disease. Digitoxin has been investigated as anti-inflammatory agent in patients with cystic fibrosis and achieved a noticeable but not statistically significant reduction in inflammation markers^56^. Acetazoleamide can cause liver injury and is associated with increased death risk in chronic liver disease patients^57^. Carbonic anhydrase CA12 is one of the targets of acetazoleamide and is expressed at low levels in healthy liver^31^. In our analysis, elevated expression of CA12 was associated with increased steatosis and NAS (high confidence observations). Sulphonamide diuretics have been used to study the link between activity of carbonic anhydrases and hepatic lipogenesis^58^. As illustrated by these examples, known drugs could be used to perturb models such as microphysiological systems or liver of animal models, gain mechanistic understanding of NAFLD and identify points of therapeutic intervention.

**Table 2:** Marketed and clinical trial drugs targeting protein products of genes in the 218-gene NAFLD progression signature or genes with genetic evidence for NAFLD. The drugs are ordered by their therapeutic application.

Drug(s)	Phase	Therapeutic application	UniProt ID of target protein(s)	Corresponding gene(s)
Obeticholic acid	Marketed	Primary biliary cholangitis	Q96RI1	NR1H4
Ursodiol				
Pioglitazone	Marketed	Antidiabetic	P37231	PPARG
Rosiglitazone				
Troglitazone				
Metreleptin	Marketed	Dyslipidemia	P48357	LEPR
Clofibrate	Marketed	Dyslipidemia	Q07869	PPARA
Fenofibrate				
Gemfibrozil				
Carvedilol	Marketed	Heart failure	P13945	ADRB3
Epinephrine				
Labetalol				
Deslanoside	Marketed	Heart failure	P05023P54710	ATP1A1FXYD2
Digitoxin				
Digoxin				
Irbesartan	Marketed	Hypertension	P30556	AGTR1
Losartan				
Valsartan				
Isosorbide dinitrate	Marketed	Vasodilators	Q02108	GUCY1A3
Nitroglycerin				
Riociguat				
Gavilimomab	Phase 3	Graft versus host disease	P35613	BSG
RA-18C3	Phase 2	Antiinflammatory	P01583	IL1A
Canakinumab	Marketed	Antiinflammatory	P01584	IL1B
Rilonacept				
Balsalazide	Marketed	Antiinflammatory	P37231	PPARG
Olsalazine				
Mesalazine				
Adalimumab	Marketed	Antiinflammatory	P01375	TNF
Etanercept				
Infliximab				
Siltuximab	Marketed	Multicentric Castleman’s disease	P05231	IL6
Capecitabine	Marketed	Cancer	P04818	TYMS
Floxuridine				
Pemetrexed				
Daunorubicin	Marketed	Cancer	P11388	TOP2A
Etoposide				
Arsenic trioxide (TRISENOX)	Marketed	Cancer	Q16881	TXNRD1
Acetazolamide	Marketed	Diuretic	O43570	CA12
Ethoxzolamide				
Nabilone	Marketed	Neuropathic pain	P21554	CNR1
Ocriplasmin	Marketed	Vitreomacular	P24043	LAMA2
		Adhesion	Q16787	LAMA3

### Candidate biomarkers for risk stratification

We identified four genes that could be evaluated as biomarkers to identify patients at risk of progression to severe NAFLD. The genes participated in the 218-gene NAFLD progression signature, encoded secreted plasma proteins and were preferentially expressed in liver (unlikely non-disease-specific and non-source-organ-specific fluctuations in biomarker levels). Decreasing mRNA levels of CYP2C19 and APOF were associated with advancing fibrosis in NAFLD (3 studies out of 3). APOF showed a high confidence association with inflammation and CYP2C19 with NAS score. Lower expression of PZP and FCN2 related to higher odds of NASH over SS in three independent studies. APOF, PZP, FCN2 and CYP2C19 were not highlighted by the authors of original publications^13–19^ as biomarker opportunities in NAFLD. Plasma APOF concentration has been suggested as fibrosis biomarker in hepatitis C^59^. An intergenic SNP rs6487679 located near PZP has been reported in association with NAFLD risk as well as elevated alanine aminotransferase^60^ and aspartate aminotransferase levels in NAFLD patients^46^.

### Signature of NAFLD progression versus clinical outcome

Genes associated with mortality or major complications in NAFLD patients could help to identify pathways for therapeutic intervention and stratify patients in need of close supervision by a physician. We were unable to locate studies investigating relationship between mRNA expression in liver on a genome-wide scale (as opposed to profiling of a small preselected set of genes) and long-term outcome in patients with NAFLD. The 218-gene NAFLD progression signature had little overlap with signatures predicting survival of patients with other liver diseases.

Dominguez *et al*. assayed hepatic expression of eleven members of CXC chemokine family in patients with severe alcoholic hepatitis and found that CXCL3, CXCL5, CXCL6 and IL18 predicted short-term mortality and related to neutrophil infiltration and portal vein hypertension^61^. The 218-gene signature contained 3 members of CXC chemokine family (CXCL6, CXCL9 and CXCL12) and transcription activator STAT1 that could modulate recruitment of neutrophils. Increasing expression of these genes was associated with worsening of NAFLD-induced fibrosis (high confidence observation).

Hoshida *et al*. published a 186-gene signature derived from liver tissue surrounding tumour and predicting mortality and liver decompensation in HCC patients^62^ and validated a 32-gene subset of this signature in NASH patients undergoing bariatric surgery^63^. We found only two genes shared between the 218-gene and 186-gene signatures. Increasing expression of CCL19 and RNASE1 was associated with advancing fibrosis in NAFLD. Both genes were linked to bad prognosis in HCC patients (Supplementary Table 2 in ref.^62^). Among genes with genetic evidence for NAFLD, SREBF2 and GCKR participated in the 186-gene signature and were linked to good outcome (Supplementary Table 2 in ref.^62^). A 122-gene hepatic stellate cell signature recently reported by the same group in association with multiple clinical outcomes in HCC and cirrhosis patients^64^ also showed a two-gene overlap: GUCY1A3 and KDELR3.

## Discussion

We derived a hepatic gene expression (mRNA) signature of NAFLD progression in adult humans through systematic meta-analysis of publicly available experiments. The signature consisted of genes whose mRNA levels had reproducible consistent-sign associations with histological traits. The main strength of our study is that we put each observation into a broad biological context (from frequently emphasized traits like fibrosis and odds of NASH over SS, to less studied NAS and progression to HCC). The replication-based approach enabled us to identify novel genes, showing potentially subtle but reproducible associations with NAFLD severity that may be non-obvious in conventional case-control design like differential expression. To the best of our knowledge, such high-resolution analysis has not been previously performed in NAFLD.

The limitations of our study were imposed by the scarcity of available data. The signature should be validated in independent studies with true longitudinal design and larger sample size. The signature could be expanded to incorporate genes associated with ballooning, insulin resistance and other comorbidity-related traits as suitable data become available. The statistical analysis was specifically adapted to handle unrelated microarray platforms, limited sample size per data set and distinct quantification systems for histology in individual data sets. Presence/absence and sign of associations represented information with compatible meaning for all models and, combined with criterion for replication, could be extracted with low risk of false positives (demonstrated in Supplementary Methods, Tables S8 and S9). The obvious limitation is lack of quantitative estimates. While we can state that mRNA expression of a given gene increases or decreases with increasing NAFLD severity, it remains an open question whether or not such relationship is strong enough for a specific application. Such questions need to be addressed in follow-up experiments and constitute directions of future work. For example, mRNA expression of APOF decreased with worsening of NAFLD-induced fibrosis. To learn whether APOF can discriminate between e.g., periportal fibrosis and bridging fibrosis, APOF should be measured on protein level with an appropriate assay in blood of NAFLD patients with the corresponding stages of fibrosis.

The 218-gene signature represents a shortlist of genes affected during NAFLD progression. Elucidation of the role of individual genes represents a direction of future work. The signature may incorporate a) potential drivers of NAFLD progression, b) genes affected during NAFLD progression but not actively driving it (down-stream events), and c) genes changing as a compensatory reaction in response to liver damage. Potential driver genes may be identified using other omics data types (e.g., methylation), tool compounds or knock-out experiments. Also, key molecular players in NAFLD may be related to mortality or major complications in NAFLD patients. We anticipate that studies on NAFLD patients, in which omics data are set in the context of phenotype (histological features, blood biomarkers etc.) and survival for the same patients, could improve our understanding of the disease.

The 218-gene NAFLD progression signature could be used to inform the choice of animal models and help to resolve issues in translational research. Hepatic gene expression (mRNA) profile of orthologue genes in a model organism under a given dietary intervention would mirror the human signature. Evaluation of similarities in gene expression profiles would complement assessment of similarities in symptoms and histological manifestations of NAFLD between humans and a given animal model.

In conclusion, NAFLD progression in human liver could be characterized by a small set of genes displaying reproducible consistent-sign associations with histological traits. This gene expression signature could be used a starting point to address current knowledge gaps on NAFLD progression.

## Electronic supplementary material

Supplementary Methods

Supplementary Data
