# Supplementary material for: Human hepatic gene expression signature of non-alcoholic fatty liver disease progression, a meta-analysis
Source: Sci Rep. 2017 Sep 27;7:12361. doi: 10.1038/s41598-017-10930-w (PMC5617840; doi:10.1038/s41598-017-10930-w)
Supplement: Supplementary file 1 — Supplementary Methods [file 41598_2017_10930_MOESM1_ESM.pdf]

Human hepatic gene expression signature of non-alcoholic fatty liver disease progression, a meta-analysis

Maria Ryaboshapkina MSc[1,*], Mårten Hammar PhD[2]
[1]Cardiovascular and Metabolic Diseases, Translational Sciences, Innovative Medicines and Early Development Biotech Unit, AstraZeneca, Pepparedsleden 1, Mölndal, 431 83, Sweden
[2]Cardiovascular and Metabolic Diseases, Translational Sciences, Innovative Medicines and Early Development Biotech Unit, AstraZeneca, Pepparedsleden 1, Mölndal, 431 83, Sweden
* maria.ryaboshapkina@astrazeneca.com

Supplementary methods

Choice of conceptual framework

There are two conceptual frameworks for integration of information from microarray experiments: meta-analysis and merging. Walsh, Hu, Batt and Santos provide the most recent and most complete overview of the topic[1]. Both approaches are equally valid. The choice of framework depends on the purpose of analysis and data situation. In meta-analysis, multiple studies are analysed separately and statistical results are subsequently integrated. By contrast, merging means combination of multiple comparable data sets into one big data set prior to statistical analysis. Comparability implies a standardized outcome such as NASH vs SS. Also, all experiments should be performed on one or several closely related microarray platforms for optimal removal of cross-platform batch-effect. Our study includes 15 data sets on 11 unrelated microarray platforms. Each data set provides a unique blend of patient characteristics (steatosis, progression to HCC etc.). Different studies use distinct quantification methods (for example, fibrosis can be given as mild vs severe fibrosis, score or grade). Hence, we adhere to the meta-analysis framework.

Outline of the meta-analysis

Microarray meta-analysis follows the typical workflow shown in Figure 1A in the review by Walsh, Hu, Batt and Santos[1]. The data sets are retrieved and prepared for down-stream analysis. A mathematical model is applied on each data set separately. Finally, statistical results are integrated across data sets. The outcome is a gene signature.

Our study follows the exact same steps. Nuances in execution of these steps are motivated by the data. We retrieve data sets that are ready for down-stream analysis (preprocessed, also known as ‘normalized’ data or Series Matrices from GEO). We use multiple end-points (fibrosis, steatosis, inflammation, NASH vs SS etc). We use linear or logistic regression models to test for association between the end-points and mRNA levels. Statistical results are regression coefficients and associated p-values. We integrate these statistical results to obtain the final gene signature in two steps. First, we identify end-points that are present in min. 3 data sets. We use such end-points to obtain intermediate gene lists (list of genes associated with fibrosis, list of genes associated with inflammation etc). Second, we merge these intermediate

gene lists and obtain gene signature of NAFLD progression. End-points represented in <3 studies serve for visual examination of results (see explanation of ‘sanity check’ and ‘negative control’ associations).

Derivation of intermediate gene lists

Individual data sets quantify the end-points differently. For example, fibrosis in NAFLD is characterized in GSE48452 (score), GSE89632 (score) and GSE49541 (advanced vs mild fibrosis). The exact quantitative interpretation of regression coefficients and p-values is different in these 3 data sets (score or two-class end-point, linear or logistic regression, different covariates in the models). The estimated coefficients and p-values are approximate (limited sample size and linear regression as a simplified model for scores). Obtaining pooled estimates for coefficients or p-values is inappropriate in our analysis settings. By contrast, all models test null hypotheses of no association between mRNA expression and an end-point. End-points are always encoded so that high values indicate severe condition and low values indicate mild condition. Hence, sign of regression coefficient has comparable meaning in all models (direction of association). Intermediate gene lists are formed by genes, whose mRNA levels either consistently increase or decrease together with worsening of a given end-point (e.g., fibrosis) in 3 independent studies.

A detailed explanation

The analysis methodology has been specifically adapted to produce robust results in the absence of true longitudinal data, despite heterogeneity of outcome variables, diverse microarray platforms, high patient heterogeneity within and across studies and small sample size per study. This Supplementary Methods section gives a detailed rationale, theoretical and empirical basis behind the approach and summarizes two benchmark experiments demonstrating method validity and reliability.

Rationale

Liver biopsies repeatedly taken from the same patients in longitudinal studies would represent ideal material to study NAFLD progression. However, to the best of our knowledge, no studies on NAFLD with longitudinal design and with a comprehensive coverage of the human transcriptome are currently available. NAFLD severity is reflected by NAS disease activity score, degree of steatosis, inflammation score and fibrosis score. Therefore, it is possible to order patients in a cross-sectional study by a given histology score from none to mild to severe and thus construct a ’pseudo time course’ reflecting increasing disease severity. Hence, relating mRNA levels to histology scores would allow to identify genes, that are affected during NAFLD progression, using cross-sectional studies.

Each available cross-sectional study features a specific subset of patients. For example, the cohort published by Moylan et al. (GSE49541) represents NAFLD patients with mild and advanced fibrosis. By contrast, the cohort by Horvath et al. (GSE61260) represents subjects with a wide range of body mass indices (BMI) and different age groups. NAFLD and NASH patients in the cohort by Horvath et al., for whom fibrosis scores are available, have either no fibrosis or mild fibrosis. Utilizing

histology scores as response variables is a way to address patient heterogeneity and unravel patterns that are non-obvious with conventional differential expression analysis between NAFLD cases and controls. For example, **Supplementary Figure S1** shows expression of DNAJC12 in relationship to diagnosis (upper row) and fibrosis severity for the same patients in the same three studies (lower row). There is a direct correspondence between the two ways to display the data only for GSE49541. In GSE49541, mRNA levels of DNAJC12 are lower in patients with advanced fibrosis than in patients with mild fibrosis. The ‘non-traditional’ view (lower row) shows that patients with severe fibrosis in GSE48452 and GSE89632 also tend to have decreased mRNA levels of DNAJC12.

Overall analysis strategy

Histology scores can be quantitative, e.g., percent of liver steatosis, or semi-quantitative in nature, for example, none, mild, moderate or severe inflammation. In an ideal data situation, one would treat a semi-quantitative histology score as an ordinal (ranked) variable and use ordinal regression. However, currently available data prohibits the use of this analysis method due to insufficient sample size per study and unbalanced representation of patients with different disease severity. Pooled analysis is also infeasible because the microarray experiments are run on different platforms and are not technically compatible (**Supplementary Table 7**). In addition, different studies use distinct scoring systems. Importantly, low values indicate mild condition and high values indicate severe condition for all scores. Hence, the scores can be used to study trends in the data. It becomes unnecessary to standardize, for example, different fibrosis scoring systems in NAFLD, as long as one considers only presence or absence and direction of association between mRNA level and fibrosis severity. Presence or absence and direction of a given trend also represent minimal qualitative information that is sufficient to obtain a gene expression (mRNA) signature.

In order to obtain this minimal information, we analyse each study and each trait separately and then identify overlap in the results.

We treat quantitative histology scores as nominal variables. We also view nominal representation of semi-quantitative histology scores as a reasonable approximation. We treat case-control outcomes such as NASH versus SS as binary variables and encode them in a consistent manner. Mild condition, for example, SS, is always encoded as 0. Severe condition, for example, NASH, is always encoded as 1. We use linear regression to investigate relationship between mRNA levels and nominal outcomes and logistic regression for binary outcomes. We adjust models for sex, age and BMI as major sources of variation, when these characteristics are publicly available on individual patient level and sample size permits estimation of a descriptive multivariate model. Other potential sources of variation, for example, medication, are not available for individual patients, are not directly incorporated in the models and hence remain ’hidden’. Measures to ensure that such hidden factors do not compromise the validity of null hypothesis test of no association between mRNA levels and histology scores are described in section **’Alternative measures to limit false positive discoveries’**, subsection ’Omitted variables and the role of ”negative control” associations’.

We reduce the amount of information, that we ask from each model, exactly to the required minimum and do not make any quantitative inference. We test the null hypothesis of no association (H_0: regression coefficient for mRNA expression = 0) versus alternative hypothesis that there is a regression slope (H_1: coefficient ≠ 0). Null hypothesis is rejected at alpha = 0.05. If null hypothesis is rejected, we register the sign of regression coefficient. Thus, each model is used only to test for absence/presence of an association and to identify direction of a trend.

A single model is sufficient to indicate absence of association (or inability to detect it, see section **'Robustness of regression analysis with respect to null hypothesis test of no association and estimated direction of regression slope'**). However, a single model is insufficient to make reliable conclusions with respect to presence of association. Microarray experiments are noisy. Therefore, we put emphasis on replication. We define NAFLD progression signature as genes, whose mRNA expression is associated with at least one histology score, e.g., inflammation severity, in 3 independent studies (see **Supplementary Table S1**, associations that are marked as 'main' in the 'association type' column). The requirement is based on data availability but is sufficient to ensure low risk of observing false positive results (see section '**Alternative measures to limit false positive discoveries**', subsection 'The role of replication in independent studies'). Also, we exclude genes, for which different probe sets show inconsistent behaviour, and make use of 'sanity check' associations with known expected behavior to further increase confidence in the observed results (see '**Alternative measures to limit false positive discoveries**', subsections "Omitted variables and the role of 'negative control' associations" and "Role of 'sanity check' associations").

Advantages and drawbacks of the methodology

Obviously, there are both advantages and caveats associated with the regression approach. On the one hand, regression analysis allows to adjust for potential sources of variation and to extract minimal required information for association analysis (absence/presence of a trend + direction) despite very limited data availability. Regression analysis is robust with respect to this minimal qualitative information. This robustness includes robustness to violations of assumption, that are typically required to produce linear and logistic models for quantitative interpretation of the data or for prediction. We discuss this topic in the next section. Adjustment for confounding variables is particularly important for identification of genes related to severity of steatosis (SS), odds of NASH over SS and NAS score, because basic demographic characteristics tend to be associated with these traits. For example, **Supplementary Figure S2** shows the relationship between LPL expression and NAS score while simultaneously taking BMI into account. Patients with high mRNA levels of LPL and high BMI tend to have high NAS scores.

On the other hand, small sample size per model covariate, the approximation of the semi-quantitative histology scores as nominal variables and skewed distributions of histology scores, such as shown in **Supplementary Figure S1**, have an impact on the precision with which regression coefficients and p-values are estimated. This negative impact on precision renders conventional approaches, that are used to limit false discoveries within a single experiment, inapplicable to our analysis settings. By conventional approaches, we mean relying exclusively on p-values and performing

adjustment for multiple testing. Also, the size of the effect, i.e., magnitude of regression coefficients, does not add valuable information for the purpose of our analysis. For example, a coefficient in a linear regression model quantifies the strength of relationship between mRNA level and a histology score. The coefficient is interpreted as 'increase by a number of units of fluorescence intensity on $\log_2$ scale, that results in one unit increase in histology score when all other covariates are held fixed'. Fluorescence intensity is a relative quantification measure (depends on instrument, microarray platform, normalization method etc). It can be used to detect trends within a given study. The absolute values of fluorescence intensity do not directly translate into mRNA concentration and are not comparable across studies. Therefore, we do not set minimal size of the effect and do not estimate minimal sample size required to demonstrate such strength of relationship.

Alternative measures to limit false positive discoveries

Model reliability depends on formal satisfaction of assumptions imposed by a given method. All general considerations for regression analysis and consequences of violation of model assumptions are well described by Kutner, Nachtsheim, Neter and Li[2]. Violation of assumptions can have consequences of different severity depending on the purpose of the analysis. In this case, we are interested in potential issues that could result in false rejection of the null hypothesis (falsely detecting an association, when there is no true association) and incorrect determination of the sign of model coefficients.

Linear relationship between predictor and outcome

Linear regression assumes that the relationship between covariates and outcome can be approximated as linear. Logistic regression assumes that the relationship between the logit link function and the covariates can be approximated as linear.

Violation of the assumption of linearity has an impact on precision with which regression coefficients are estimated. Mild non-linearity should not have an impact on the null hypothesis test and sign of association. Severely non-linear relationships should not be detected (false negative result). In the strict mathematical sense, proof of these two statements requires a separate simulation study. Instead, we simply demonstrate, that nonparametric methods agree on presence/absence and direction of association with regression models (see next section). We also explain cases when the methods disagree. Nonparametric alternatives to regression analysis (Kendall tau b and Wilcoxon rank sum test) do not make the assumption of linearity,

Assumption of normality and sample size

Linear regression assumes that model residuals (the 'error terms') are approximately normally distributed. Residuals of a logistic regression model follow binomial distribution.

In linear regression, the assumption of normality is made because the test statistic for null hypothesis test (H_0: regression coefficient = 0) follows t-distribution. Practice shows that the test statistic and p-values are roughly accurate even when the distribution of residuals departs from the normal distribution. In particular, see[1]

Chapter 2, section 2.3 'Departure from normality and inference'. Approximately normal distribution of residuals in a linear regression model can arise when predictor(s) and/or outcome are not normally distributed.

Testing for normality with numeric tests (for example, Jarque-Berra skewness and kurtosis test) has limited value for small samples. Instead, we include studies with at least 15 patients into the analysis. This is an empirical rule of thumb (see Minitab 17 white paper on multivariate linear regression for details of the simulation study, available online at http://support.minitab.com/en-us/minitab/17/Assistant_Multiple_Regression.pdf). It helps to create a data situation, in which the test for null hypothesis of no association is likely to be valid.

Independence of residuals

Residuals of a regression model can be correlated when a data set includes, for example, technical replicates or samples taken from the same patient within short time interval. If the assumption of independence is violated, the standard error in estimating the regression coefficients is lower than the 'true' standard error. This can lead to false rejection of null hypothesis (false positive result). Liver samples come from distinct patients in the studies that we have included into our analysis. Therefore, assumption of independence is not violated. GSE33258 does contain technical replicates. However, close examination of experimental design shows that this is a two-channel microarray experiment. Technical replicates are created by exchanging normal patient material on the reference channel (fibrosis patient 1 vs normal patient 1, fibrosis patient 1 vs normal patient 2, etc). Post-bariatric surgery samples in GSE48452 have been taken 5 to 9 months after the procedure[3], i.e., when sufficiently long time has elapsed from the first biopsy to the repeated biopsy. Therefore, the samples can be assumed to be independent for the purpose of our analysis.

Homoscedasticity and influential observations

Homoscedasticity means that variance of model residuals is equal for all levels of the predictor or predictors. For example, the variance of error terms does not decrease or increase with increasing mRNA levels. Influential observations are data points, in our case, patients, that have such extreme values of predictor and/or outcome that they can change coefficient estimates ('pull the regression line towards themselves'). Strong outliers are examples of influential observations. Violation of the assumption of homoscedasticity and presence of influential observations have an impact on precision with which coefficients are estimated, but are unlikely to compromise the validity of null hypothesis test of no association or lead to incorrect determination of the sign of the regression coefficients. Formal proof of these statements is a separate topic and is outside of the scope of our investigation. An indirect proof of the statements is given in the next section. Nonparametric methods do not make homoscedasticity assumption and operate on the basis of ranks, rather than the actual values, and are thus less affected by observations with extreme values.

Multicollinearity

Multicollinearity is a situation when the predictor of interest, in our case, mRNA level, can be reasonably well predicted in linear fashion based on other covariates, in our case, demographic characteristics. Multicollinearity can have negative consequences for multivariate linear and logistic regression models. Strong multicollinearity with respect to mRNA expression can inflate standard error of the corresponding regression coefficient and lead to an unstable coefficient estimate. The coefficient can even be estimated with a wrong sign. Thus, the most likely consequence of multicollinearity is a false negative (detecting absence of association, when there actually is an association).

Omitted variables and the role of 'negative control' associations

'Hidden' variables are characteristics that are not available on individual patient level or available in too few data sets to consistently treat them as potential confounding variables. Common comorbidities of NAFLD like diabetes or dyslipidemia, may have an impact on mRNA levels and on histology scores. Omitting them from a model has no impact on the validity of null hypothesis test as long as there is no association between mRNA levels and diabetes and dyslipidemia-related traits (variables can be assumed to be independent). We use these traits as 'negative control' associations for visual examination of association profiles of the signature genes (see **Supplementary Table S1**). Predominantly white columns indicating no association between signature genes and diabetes- related and dyslipidemia-related traits in Figures 2 and 3 in Results section of the manuscript show that these omitted variables do not compromise the validity of the results.

The role of replication in independent studies

Replication in independent studies decreases the possibility to observe false positive associations between mRNA levels and histology scores due to numeric artifacts, technical reasons and 'hidden' variables. Numeric artifact are patterns that are detected due to a specific distribution of values in the data (skew, influential observations, etc). Technical artifacts are issues, that are caused by unspecific hybridization on a microarray chip. The distributions of 'hidden' variables such as ethnic origin, diet or medication are unlikely to coincide in different cohorts.

Detection of consistent-sign association is three independent studies is sufficient to be confident that the results do not represent a coincidence. To illustrate, we conduct a simulation experiment. We take complete expression matrices (all samples and all probe sets) for the studies, that form the core of our analysis, randomly shuffle the histology scores in each study and perform regression analysis for 24 traits, that are marked as 'main' in **Supplementary Table S1**. We repeat the procedure 1,000 times. In each iteration, we count the number of genes showing consistent-sign associations to a given histology score in three independent studies. Simulation outcome in **Supplementary Table S8** indicates that the NAFLD-progression signature, reported in Figures 2 and 3 in main text, is very unlikely to arise by chance.

Role of 'sanity check' associations

In addition, we use ’sanity check’ associations (see **Supplementary Table S1**). The term is borrowed from computer science and refers to simple numeric measures, that are used to aid visual examination of results and indicate potential issues with the data or errors in the code.

’Sanity checks’ have known expected behaviour. For example, elevated levels of liver injury markers such as alanine transaminase are widely used in clinical practice to identify patients with liver disease. If mRNA level of a given gene is associated with severity of liver injury (NAS, inflammation, fibrosis), observing same-sign association with alanine transaminase adds confidence. By contrast, behaviour of ’sanity checks’, that goes against the state-of-the art knowledge of liver biology, is an indicator of potential problems.

The ’sanity checks’ and ’negative controls’ carry two more important functions. First, they participate in clustering of the association profiles together with the ’main’ associations and help to answer the question: ’Are there patterns in the data, that can be identified by visual inspection of the results?’ Second, the transcriptomics data resources for NAFLD are currently very scarce compared to other disease areas like asthma or oncology. We would like to encourage other scientists to use our work, extend the analysis when more data becomes available or generate hypotheses for other experiments. Therefore, we deliberately avoid selective reporting and instead display data despite the fact that the ’sanity check’ and ’negative control’ traits have been assessed in fewer than 3 studies and thus are not part of the main observations.

Robustness of regression analysis with respect to null hypothesis test of no association and estimated direction of regression slope

The previous section is dedicated to theoretical considerations that form the basis of our analysis. In practice, are conclusions made with respect to presence/absence of association and sign of association reliable?

If an association or lack thereof is true, a conceptually different method should give the same result.

Nonparametric methods make different assumptions about the data. Kendall tau b is a nonparametric alternative to univariate linear regression. Kendall tau b is a correlation coefficient that can be used to measure strength of a monotonic (not only linear) relationship between a continuous and/or ordinal predictor and a continuous and/or ordinal outcome. The method takes ties into account (patients with exact same value of a given histology score). The null hypothesis H_0: Kendall tau b = 0 is rejected in favour of alternative hypothesis H_1: Kendall tau b ≠ 0 at alpha = 0.05. The sign of association is the sign of the correlation coefficient.

Wilcoxon rank sum test is a nonparametric alternative to univariate logistic regression. Wilcoxon rank sum test assumes that predictor is nominal or ordinal in nature and that samples in groups A and B, that are being compared, are independent. The null hypothesis H_0: ’the distributions of predictor in A and in B are not different’ is rejected in favour of alternative hypothesis H_1: ’there is a difference in

distributions' at alpha = 0.05. We take sign of the difference between median predictor values in B and A as sign of the association.

As mentioned previously, Kendall tau b and Wilcoxon rank sum test are approaches for univariate analysis, i.e., they do not allow for adjustment for potential confounding variables. Hence, we expect some disagreement between output from regression models and their nonparametric counterparts. In particular, we expect high disagreement for associations between mRNA levels and degree of steatosis, NAS and odds of NASH over SS.

We have performed associations analysis with nonparametric methods for all genes and traits reported in supplementary tables 2, 5 and 6. Kendall tau b and Wilcoxon rank sum test are implemented in R base functionality. **Supplementary Table S9** shows that regression models and their nonparametric alternatives mostly agree with respect to presence/absence and sign of association.

Supplementary Table S9 summarizes the overall picture. For the genes constituting the 218-gene signature, the disagreement for distinct traits ranges from 0% to 21.6%. with interquartile range 2.8%-7.1% and median value of 5.1%. We have observed the lowest disagreement rate of 0% for the following traits: fibrosis progression in portal tract tissue in GSE33650, ballooning and mild versus no fibrosis in GSE61260 and enlarged liver in GSE61376. Almost all models report absence of association for these traits. The sample size for each of these four traits is below 20. These results probably represent true negative findings. There are biological reasons to expect very minor changes in mRNA levels, that are unlikely to be reliably detected given the amount of data. For example, ballooning affects only few hepatocytes while the mRNA levels are measured on bulk tissue samples. We also observe low disagreement for inflammation severity (0.5%), amount of eicosapentaenoic (0.5%) and docosahexaenoic acids (0.9%) in liver in GSE89632. In these cases, sample sizes are relatively large (43, 52 and 52) and the distributions of outcome are well approximated as continuous (inflammation) or are truly continuous (% of fatty acids). Univariate analysis shows that the levels of fatty acids are confounded by BMI. Apparently, this confounding effect does not influence the detected associations between mRNA levels and levels of fatty acids, because univariate nonparametric methods report almost exactly the same results. By contrast, regression analysis and nonparametric methods show the highest disagreement for fibrosis severity (21.6%), degree of steatosis (20.6%) and NAS score (20.2%) in GSE48452. This data set contains a subset of patients after bariatric surgery. Univariate analysis shows that there is a relationship between bariatric surgery status and fibrosis, steatosis and NAS scores in GSE48452. We also detect a relationship between NAS score and BMI. GSE48452 is one of the key studies, that we use to derive the NAFLD progression signature. Hence, many genes would not have been captured if we used univariate nonparametric methods to obtain the signature. Still, parametric and nonparametric method reach perfect agreement for 151 out of 218 signature genes (69.3%) (**Supplementary Table S10** in Excel file).

In summary, we demonstrate that parametric methods are adequate and robust for the purpose of our analysis, and, in combination with the requirement for replication, are suited to control false positive discoveries.

Notes on the meta-network

We use regression models to search through ~20,000 human genes and identify the 218-gene signature of NAFLD progression. mRNA expression of each gene in this signature is associated with one or more aspect of NAFLD progression (inflammation, steatosis, progression to HCC etc.). The literature-derived set of 62 genes with genetic evidence for NAFLD also represents special interest. Each of these 62 genes has a NAFLD-associated SNP within the gene boundaries or in immediate vicinity of the gene on the chromosome. These 218 + 62 genes form a subset of interest. We use co-expression analysis to investigate the relationships between mRNA expression of all pairs of genes within this subset.

The meta-network can be contemplated as a data-driven and easy-to-visualise alternative to pathway enrichment analysis. Co-expression illustrates patterns or 'biological themes' in the data. Correlation may point to a variety of different relationships between genes such as co-localization (subunits of a protein complex, common cell type) or a common biological process (e.g., DNA repair). The advantage is that the correlation patterns originate from measurements on tissue and disease of interest. The method does not rely on available literature-derived annotation and has no bias towards prior knowledge.

Co-expression networks are based on pairwise correlations between mRNA levels. Correlation is a sensitive instrument and it also captures experimental noise. Hence, we require correlation patterns to be reproduced in at least 3 independent studies out of 5 NAFLD studies with total sample size >30 (empirical rule of thumb). We use hard-threshold. We want to eliminate weak correlations that most probably represent experimental noise, unspecific hybridization etc. For example. the smallest study for meta-network construction has N = 44 samples (GSE33814). We can use asymptotic distribution because 44 > 30: $t_{N-2} = \frac{rho * \sqrt{N-2}}{\sqrt{1-rho^2}}$, where rho is Spearman correlation. Critical value for 2-tailed test on t-distribution with df = 44-2 and alpha = 0.05 is 2.02. Thus, theoretical threshold for rho is 0.3. Microarrays are noisy. We need to obtain an objective and data-driven threshold (which is typically higher than the threshold based on asymptotic distribution). We use pickHardThreshold function from WGCNA package. This function is based on the fit to scale-free topology model. An excellent review of the topic of network properties of biological systems in general and scale-free topology in particular is published by Barabási and Oltvai[4]. We are justified to use the scale-free topology model because genes in our 218 + 62 subset of interest come from different sources. We expect some but not all of them to belong to the same 'pathway'. We identify threshold = 0.53 as illustrated in **Supplementary Figure S3**.

Footnote

3D plots in Supplementary Figure S2 are produced with plot3D package in R[5].

References

1. Walsh, C. J., Hu, P., Batt, J. & Santos, C. C. Microarray Meta-Analysis and Cross-Platform Normalization: Integrative Genomics for Robust Biomarker Discovery. *Microarrays (Basel)* **4**, 389-406, doi:10.3390/microarrays4030389 (2015).
2. Kutner, M., Nachtsheim, C., Neter, J. & Li, W. *Applied linear statistical models*. (McGraw Hill, 2004).
3. Ahrens, M. *et al.* DNA methylation analysis in nonalcoholic fatty liver disease suggests distinct disease-specific and remodeling signatures after bariatric surgery. *Cell Metab* **18**, 296-302, doi:10.1016/j.cmet.2013.07.004 (2013).
4. Barabasi, A. L. & Oltvai, Z. N. Network biology: understanding the cell's functional organization. *Nat Rev Genet* **5**, 101-113, doi:10.1038/nrg1272 (2004).
5. Soetaert K. (2016). plot3D: Plotting Multi-Dimensional Data. R package version 1.1. URL: https://CRAN.R-project.org/package=plot3D

Supplementary Table S7. Detailed information about microarray platforms used in our study. We have 15 data sets on 11 distinct platforms (see Platform ID) from 5 manufacturers. The microarray platforms were not closely related (see Technology column).

Data set	Made available on GEO	Platform ID	Manufacturer	Technology
GSE48452	Aug 08, 2013	GPL11532	Affymetrix	exon and gene level array
GSE61260	Nov 03, 2014	GPL11532	Affymetrix	exon and gene level array
GSE89632	Nov 08, 2016	GPL14951	Illumina	beadchip
GSE59045	Jul 02, 2015	GPL15207	Affymetrix	PrimeView array, 3’ array but different pobe set design than e.g. GPL570
GSE49541	Aug 30, 2013	GPL570	Affymetrix	3' array
GSE15653	Jun 01, 2009	GPL96	Affymetrix	3' array
GSE33814	Dec 10, 2012	GPL6884	Illumina	beadchip
GSE33258	Nov 21, 2011	GPL14795	Operon Biotechnologies	spotted oligonucleotide array
GSE33650	Aug 13, 2013	GPL14877 (analogue to GPL570)	Affymetrix	3' array
GSE11536	May 24, 2008	GPL5215	INSERM	spotted DNA/cDNA (custom-made array)
GSE84044	Aug 21, 2016	GPL570	Affymetrix	3' array
GSE61376	Sep 13, 2014	GPL6947	Illumina	beadchip
GSE6764	Jun 21, 2007	GPL570	Affymetrix	3' array
GSE54238	Jan 22, 2014	GPL16955	NimbleGen	high density DNA array
GSE14323	Jan 08, 2009	GPL571	Affymetrix	3' array

Supplementary Table S8. Distributions of the number of genes showing consistent-sign associations with a given histology trait in 1,000 random permutations in comparison with the obtained NAFLD progression signature.

Category	Consistent-sign association in 3 independent studies with …					
	… >= 1 NAFLD-related trait	… >=2 NAFLD-related traits	… only degree of SS	… only NAS score	… only odds of NASH vs SS	... only fibrosis severity in NAFLD
N chance findings in 1,000 permutations as median and empirical 95% confidence interval (in brackets)	3 (0-14)	0 (0-0)	0 (0-4)	0 (0-5)	0 (0-4)	0 (0-9)
N genes in NAFLD-progression signature	218	57	7	18	32	104
N permutations in which the number of chance findings was at least as large as the number of signature genes	0	0	13	1	1	0
Empirical p-value (Probability that N chance findings >= N signature genes)	0	0	0.013	0.001	0.001	0

Supplementary Table S9. Agreement between regression models and their nonparametric alternatives.

% of cases in which parametric method and its nonparametric alternative…		All genes in Suppl. Tables 2, 5 and 6	Suppl. Table 2, genes in 218-gene signature (Figs. 2 and 3 in text)	Suppl. Table 2, genes with genetic associations to NAFLD (Fig. 1 in text)	Suppl. Table 5, HCV	Suppl. Table 6, HCC
Disagree	…disagree on presence/absence and/or sign of association	4.3	5.6	4.3	3.9	4.1
Agree	…both indicate no association	76.4	66.8	79.5	78.0	78.5
	…both indicate presence of association and agree on the sign of association	19.0	27.2	15.9	17.7	17.2
	…both report inconsistent-sign associations for different probe sets	0.3	0.4	0.3	0.4	0.2
	Total agreement	95.7	94.4	95.7	96.1	95.9

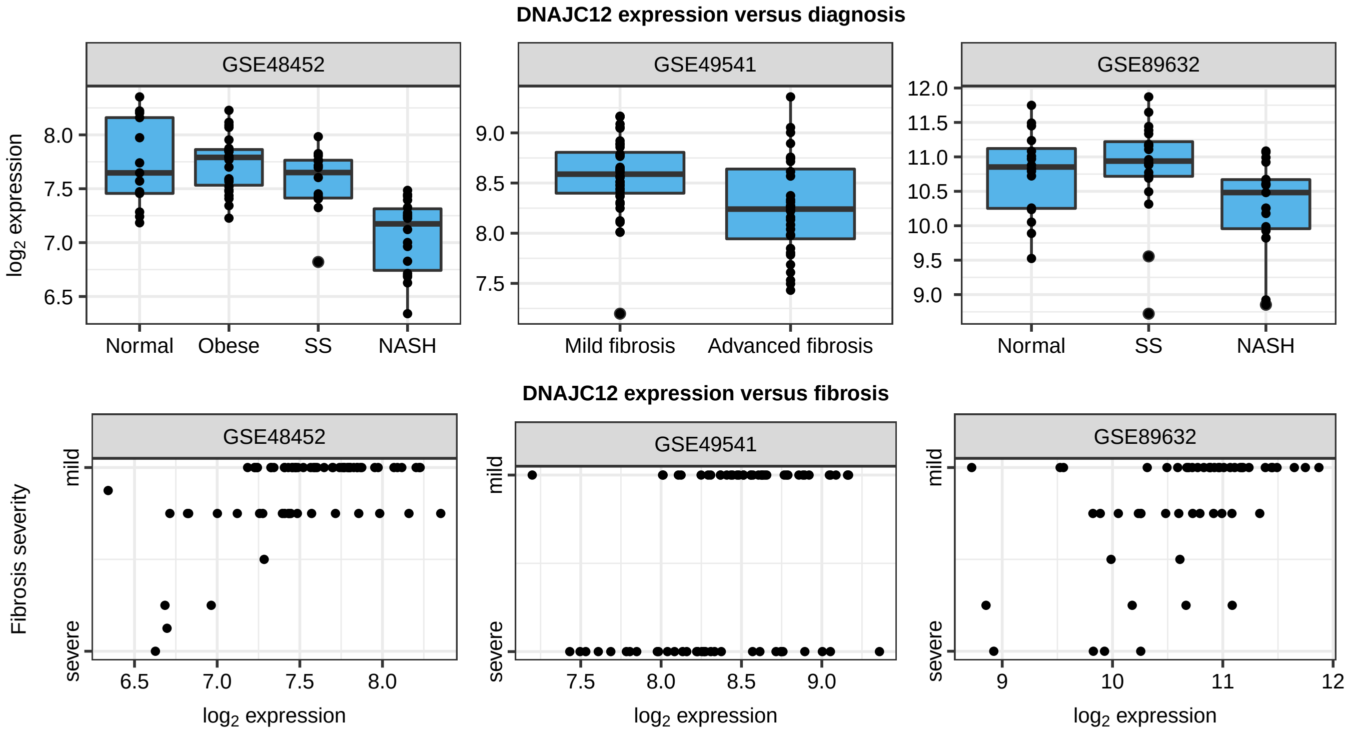

Supplementary Figure S1. mRNA expression of DNAJC12 in relationship to diagnosis (upper row) and fibrosis severity (lower row) in GSE48452, GSE49541 and GSE89632. mRNA expression is shown for the following probe sets: 218976_at in GSE49541, 7933933 in GSE48452 and ILMN_1725773 in GSE89632.

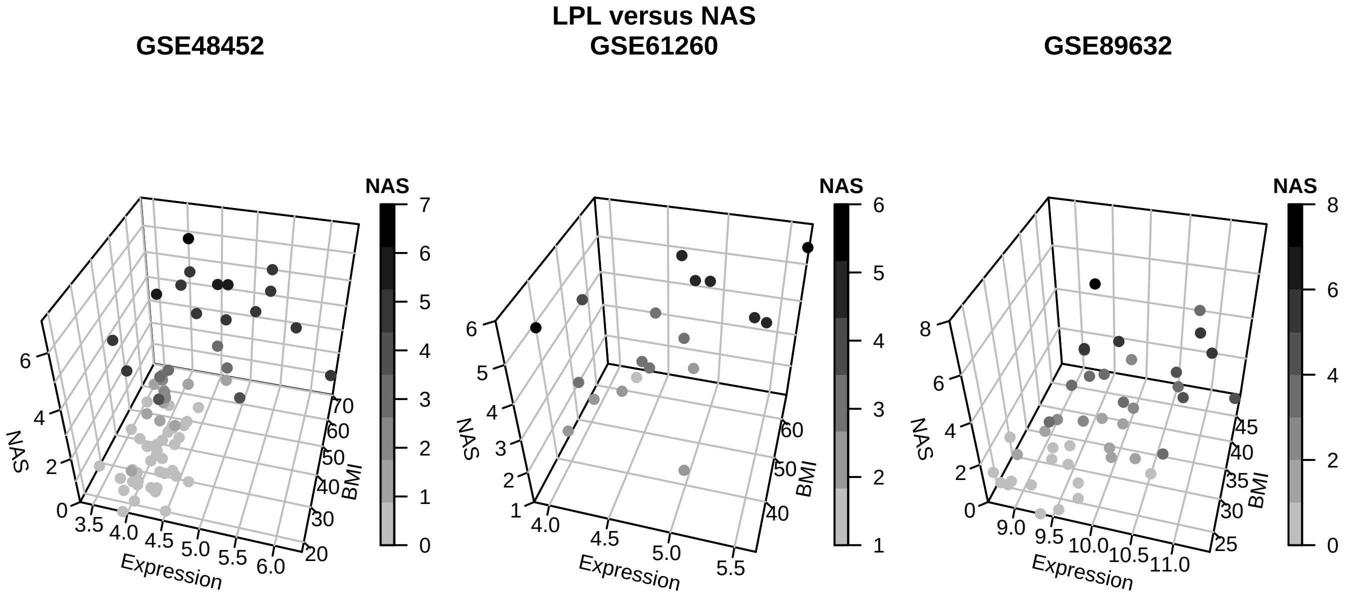

Supplementary Figure S2. mRNA expression of LPL in relationship to NAS score and BMI in GSE48452, GSE61260 and GSE89632. The plot for GSE61260 displays values for a subset of NAFLD and NASH patients. The plots for GSE48452 and GSE89632 also incorporate control patients. mRNA expression levels are shown on $\log_2$ scale and represent the following probe sets: 8144917 in GSE48452 and GSE61260, and ILMN_1786444 in GSE89632.

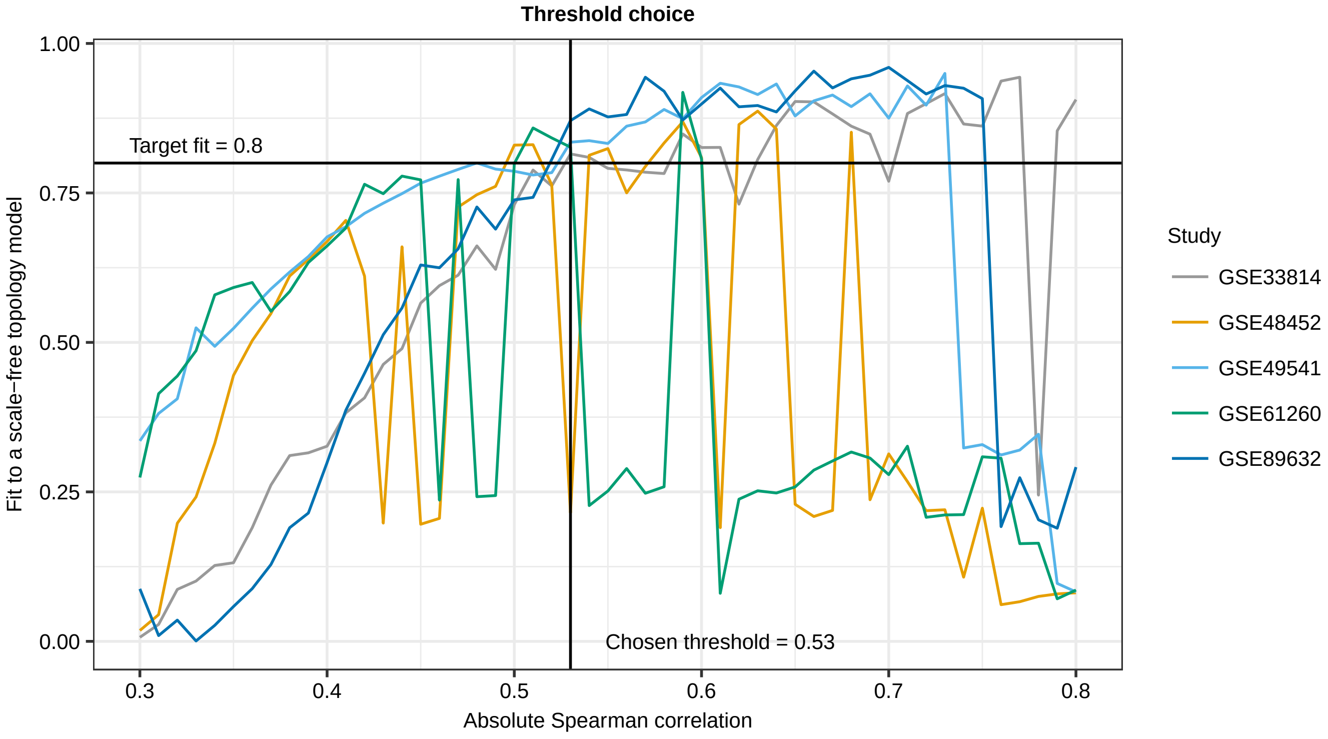

Supplementary Figure S3. Choice of hard threshold for construction of the meta-network. The threshold value for Spearman correlation is chosen on absolute scale. Each line represents R^2 fit to a scale-free topology model, that is calculated at each correlation threshold with step of 0.01. The chosen threshold corresponds to the smallest value at which 4 out of 5 individual networks display approximate scale-free topology.
